# What Is Ageism? Do Our Measures Tell Us?

**DOI:** 10.1093/geroni/igaa057.2223

**Published:** 2020-12-16

**Authors:** Jerin Lee, Jenna Wilson, Alexandria Ebert, Natalie Shook

**Affiliations:** 1 University of Connecticut, Storrs, Connecticut, United States; 2 West Virginia University, Morgantown, West Virginia, United States

## Abstract

Ageism has been operationalized in a number of ways over the past half century, and several measures of ageism have been developed to reflect these conceptualizations. This presentation evaluates the extent to which existing ageism measures capture similar or different aspects of ageism. We examined the construct validity of several ageism measures in a sample of 473 undergraduate students (Mage = 19.5 years). Participants completed an online survey of ageism measures, as well as measures of convergent (e.g., aging semantic differentials, attitudes toward various age groups) and discriminant validity (e.g., social dominance orientation, attitudes toward other social groups, sexism). Ageism measures were generally positively associated with one another, though correlations were mostly weak to moderate in magnitude. There was also weak evidence of convergent and discriminant validity. These findings demonstrate conceptual problems with current ageism measures as they do not appear to reflect a common construct.

